# Providing Task Instructions During Motor Training Enhances Performance and Modulates Attentional Brain Networks

**DOI:** 10.3389/fnins.2021.755721

**Published:** 2021-12-09

**Authors:** Joaquin Penalver-Andres, Karin A. Buetler, Thomas Koenig, René Martin Müri, Laura Marchal-Crespo

**Affiliations:** ^1^Motor Learning and Neurorehabilitation Laboratory, ARTORG Center for Biomedical Engineering Research, University of Bern, Bern, Switzerland; ^2^Psychosomatic Medicine, Department of Neurology, Inselspital, Bern University Hospital, University of Bern, Bern, Switzerland; ^3^Translational Research Center, University Hospital of Psychiatry and Psychotherapy, University of Bern, Bern, Switzerland; ^4^Gerontechnology and Rehabilitation Group, ARTORG Center for Biomedical Engineering Research, University of Bern, Bern, Switzerland; ^5^Perception and Eye Movement Laboratory, Department of Neurology and BioMedical Research, University of Bern, Bern, Switzerland; ^6^Department of Neurology, Inselspital, Bern University Hospital, University of Bern, Bern, Switzerland; ^7^Department of Cognitive Robotics, Delft University of Technology, Delft, Netherlands

**Keywords:** motor learning and control, cognitive neuroscience, neural biomarkers, variability, attention/working memory, EEG oscillations, instructions and feedback

## Abstract

Learning a new motor task is a complex cognitive and motor process. Especially early during motor learning, cognitive functions such as attentional engagement, are essential, e.g., to discover relevant visual stimuli. Drawing participant’s attention towards task-relevant stimuli—e.g., with task instructions using visual cues or explicit written information—is a common practice to support cognitive engagement during training and, hence, accelerate motor learning. However, there is little scientific evidence about how visually cued or written task instructions affect attentional brain networks during motor learning. In this experiment, we trained 36 healthy participants in a virtual motor task: surfing waves by steering a boat with a joystick. We measured the participants’ motor performance and observed attentional brain networks using alpha-band electroencephalographic (EEG) activity before and after training. Participants received one of the following task instructions during training: (1) No explicit task instructions and letting participants surf freely (implicit training; IMP); (2) Task instructions provided through explicit visual cues (explicit-implicit training; E-IMP); or (3) through explicit written commands (explicit training; E). We found that providing task instructions during training (E and E-IMP) resulted in less post-training motor variability—linked to enhanced performance—compared to training without instructions (IMP). After training, participants trained with visual cues (E-IMP) enhanced the alpha-band strength over parieto-occipital and frontal brain areas at wave onset. In contrast, participants who trained with explicit commands (E) showed decreased fronto-temporal alpha activity. Thus, providing task instructions in written (E) or using visual cues (E-IMP) leads to similar motor performance improvements by enhancing activation on different attentional networks. While training with visual cues (E-IMP) may be associated with visuo-attentional processes, verbal-analytical processes may be more prominent when written explicit commands are provided (E). Together, we suggest that training parameters such as task instructions, modulate the attentional networks observed during motor practice and may support participant’s cognitive engagement, compared to training without instructions.

## Introduction

Motor learning is a complex cognitive and motor process leading to relatively permanent behavioural and neural changes (i.e., brain plasticity; Krakauer, 2006). Fitts (1964) defines three stages of motor learning: the *cognitive, associative*, and *autonomous* stages. In the early *cognitive* and *associative* stages—where task rules are inferred and appropriate sequences of actions determined and refined—cognitive engagement (e.g., attention) is essential for motor learning. Attention plays a critical role in discovering relevant visual stimuli (selective visual attention) and generating effective and controlled responses (executive attention). However, real-life training scenarios present vast amounts of simultaneous stimuli (auditory, visual, etc.), which compete for the trainees’ limited attentional resources. While motor learning is considered to rely on implicit/procedural processes (Shea et al., 2001; Vidoni and Boyd, 2007), coaches can help trainees focusing their attention on task-relevant stimuli, typically, using instructions.

It has been shown that, when faced with the same motor task, proficient performers (in the autonomous stage) tend to manifest more extensive, associated, and detailed explicit knowledge about the given task than low skilled performers in the cognitive and associative stages—e.g., during ball throwing (Martini and Shore, 2008), and tennis practice (McPherson and Thomas, 1989). This cognitive advantage generally results in an enhanced motor performance—e.g., during tennis practice (Del Villar et al., 2007) or golf putting (Masters, 1992). Providing task-related explicit knowledge about the task rules to trainees during motor training seems to accelerate motor learning in ecological—e.g., in golf-putting (Hardy et al., 1996)—and computer-simulated motor tasks—e.g., in an anticipation-coincidence task (Albinet and Fezzani, 2003). For instance, using explicit written instructions about the correct movement patterns required to master golf putting swing enhances motor performance during the first stages of learning (Hardy et al., 1996). Further, trainees with tactical knowledge before the task training starts—e.g., attack and defence tactics in soccer (Cardoso et al., 2019)—show reduced cognitive effort when taking game-related decisions, compared to trainees without prior tactical knowledge. Therefore, providing explicit knowledge of the task rules may help participants to draw their limited attentional resources towards task-relevant stimuli and boost their motor performance (Albinet and Fezzani, 2003).

Apart from providing (e.g., written) explicit knowledge of the task rules, providing visual cues during training may help drawing participants’ attention towards the task features/stimuli relevant to master the skill (Poulter et al., 2005; Wolpert et al., 2011). For example, participants trained to identify soccer penalty kick patterns using visual cues—e.g., highlighting postural hallmarks of the penalty kicker—showed more task-relevant eye fixations and recalled more features regarding these optimal patterns (Poulter et al., 2005). Importantly, in a more recent study, D’Innocenzo et al. (2016) found that the visuo-attentional advantages associated with visual cues enhance motor learning—i.e., golf trainees who were provided with visual cues during golf swing practice outperformed trainees who trained without visual cues.

Together, studies have shown that instructions—using a combination of explicit commands and visual cues to enforce task rules during training—support motor performance and learning (Janelle et al., 2003), potentially by supporting early cognitive engagement. However, to date, less is known about the influence of visual or explicit written task instructions on attentional brain networks. Nevertheless, knowing how task instructions could be provided to optimally support early cognitive-attentional processes may be crucial to design better training routines. For example, the engagement of motor learning-related attentional networks could be enforced by instructions that explicitly inform participants about the underlying task rule, or by visually cueing relevant stimuli during training, allowing participants to learn the task rule more implicitly.

In this study, we aim at investigating the effect of providing task instructions in written or using visual cues during training on motor performance and attentional brain networks using *electroencephalography (EEG).* Alpha-band strength is a promising neural marker of various types of attentional processes involved during motor learning. More efficient visuo-attentional processes are linked to a decrease in cortical activity over posterior brain areas (Lee et al., 2012), as reflected in increased EEG alpha-band power (7–15 Hz) (Palva and Palva, 2007; Del Percio et al., 2009; Klimesch, 2012). Further, researchers have consistently reported neural modulations of the amplitude and topographical distribution of EEG alpha-band activity during cognitive tasks (Albares et al., 2014) when attention is refocused by using instructions—e.g., with visual cues (Haegens et al., 2012; Payne et al., 2013; Schneider et al., 2019) or explicit written instructions (Samaha et al., 2018; van Duijn et al., 2020). In particular, when a motor task involves visuo-attentional processes—e.g., when visual cues are provided —, alpha-band activity modulation over parieto-occipital areas has been observed (Payne et al., 2013). Likewise, in sports settings (e.g., golf or table tennis), when verbal working memory is required—e.g., when explicit verbal instructions are provided —, EEG alpha-band activity over frontal and temporal areas is modulated (Zhu et al., 2011a; Buszard et al., 2016).

So far, the alpha-band related EEG-based studies on visuomotor attentional processes have focused on: (1) simple stimulus-response paradigms analysing electrode-level event-related spectral changes (Thut et al., 2006; Haegens et al., 2012; Payne et al., 2013; Albares et al., 2014; Schneider et al., 2019) or (2) discrete complex motor tasks (e.g., laparoscopic surgery or table-tennis; Zhu et al., 2011a; Buszard et al., 2016) analysing electrode-level spontaneous activity. Concretely, electrophysiology studies showed that neural firing occurs at troughs of the alpha-band waves (Haegens et al., 2011; Klimesch, 2012), relating increased alpha-band power with periods of low neural excitability, which support attentional and working memory processes (Palva and Palva, 2007; Klimesch, 2012). Despite the fact that changes in particular attentional demands are reliably associated with alpha changes at particular scalp sites, the functional role of local (electrode-level) alpha-band strength modulations is ambiguous and generally oversimplified in the literature (Palva and Palva, 2007; Klimesch, 2012). However, literature investigating attentional networks (i.e., event-induced scalp-wide spatio-temporal dynamics) during complex visuomotor tasks is missing. In this study, we conducted a spatiotemporal analysis of event-elicited scalp electric fields (Murray et al., 2008). In doing so, we avoided the methodological limitations of the “traditional” event-related potential (ERP) analyses, which usually focus on known components of interest occurring at predefined sites and periods of interest (Murray et al., 2008). Spatio-temporal analysis methods make it possible to identify the different electrical scalp-field spatial and temporal properties related to different experimental conditions (Michel et al., 2009), supporting researchers to pinpoint statistically different cortical generators or networks that produced the condition-specific electric scalp fields (Michel et al., 2009; Koenig et al., 2011).

With this work, we aimed to shed some light on the scalp-wide modulation of attentional brain processes and resulting motor performance changes after training with different task instructions. We ran an experiment with 36 healthy participants who trained a complex visuomotor task [i.e., a task with high demands on attention, memory, and processing capacity together with motor execution (Wulf and Shea, 2002; Basalp et al., 2021)]: to surf a virtual boat on waves as fast as possible using a joystick, i.e., an attention and planning task. Participants were randomly allocated to three different training groups that differed on the received task instructions enforcing different task-relevant stimuli: (1) letting the participants surf freely (implicit, IMP), (2) providing them with written instructions on how to correctly align the boat with the wave (explicit, E); and (3) implicitly instructing them to move correctly using visual cues (explicit-implicit, E-IMP). We hypothesized that providing task instructions (i.e., in the E and E-IMP groups) would support attention (i.e., the ability to perceive task-relevant stimuli) during training, enhancing motor performance pre-post training. Such attentional facilitation would be reflected in topographic pre-post training changes of increased alpha-wave strength and better motor performance after training than practicing without task instructions (IMP group). Finally, we expected to observe a generalization of the skills gained during training (Schmidt and Lee, 2011) when E and E-IMP participants are challenged with a different task: avoiding obstacles while surfing waves, i.e., a reaction task. Although the results of this work do not have a direct implication for surfing/sailing training, our investigation contributes to gaining a better understanding of the potential neural and behavioural benefits of enforcing task-relevant stimuli using visual cues or written commands in applications where instructions are commonly used (e.g., sports training and neurorehabilitation).

## Materials and Methods

### Participants

Thirty-six healthy volunteers (14 women, aged 20–59 years, μ_*age*_ = 27.9 yrs.; σ_*age*_ = 6.64 yrs.; gender and age balanced across groups, *p > 0.05*) participated in the study. Thirty-one participants were right- and five participants left-handed as assessed by the Edinburgh Handedness Inventory (Oldfield, 1971). All participants were naive to the virtual surfing task. Up to 38 % of participants reported prior experience with virtual reality (VR), and 41 % had experience in video gaming. One participant had a sailing license and three more had some experience with sailing. Previous experience with VR, video gaming, and sailing was balanced across groups (*p > 0.05*).

All participants provided written informed consent before participation in the study. The studies involving human participants were reviewed and approved by the local Ethics Committee (ref.: 2018-01179) and the Swiss Agency for Therapeutic Products (Swissmedic ref.: 10000432). The study is registered in ClinicalTrials.gov (NCT04759976) and EUDAMED (CIV-19-01-026764) under the title “Optimize motor Learning to Improve Neurorehabilitation” (“OnLINE”). No potentially identifiable human images or data are presented in this study.

### Experimental Setup

**FIGURE 1:**
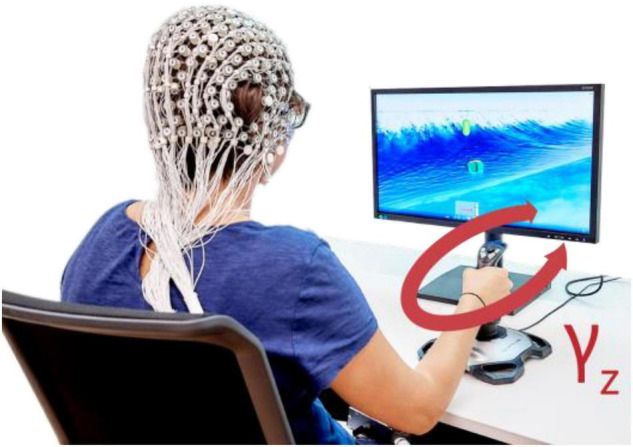
Experimental setup: participants sat comfortably on a chair and controlled a virtual surfing boat by rotating a joystick. The rotation of the joystick is denoted as γ_**z**_.

Participants sat comfortably on a chair, resting their chin on a chin rest while performing the virtual surfing task developed in Unity (*Unity Technologies, United States*). They controlled the orientation of the virtual boat by rotating the vertical axis of a joystick (mechanical angle limits: [−15°, 15°]; model: J-UK-17; *Logitech, Switzerland*) with their dominant hand (Figure 1). The height and position of the chin rest (not visible in Figure 1), chair, joystick, and computer screen were adapted to participants’ hand dominance and controlled across participants. Participants’ neural activity was recorded using a 256-channel Hydrogel cap and EGI Net Amps amplifier (*Electric Geodesics, United States*). EEG data acquisition was synchronized with the virtual surfing task via a parallel port.

### Virtual Surfing Environment

To address our research question, we required a comparably complex visuomotor task that resembles a real-life scenario. We sought a novel lab-based virtual motor task that allowed us to repeat and record many EEG trials during practice and perform event-related spatio-temporal analysis with enough statistical power. For this, we needed to keep participants engaged during the complete duration of the experiment. As such, we required a challenging and cognitively engaging real-life task to ensure a long learning curve of a complex motor task with a cognitive component: an underlying task rule (“aligning the boat to the direction orthogonal to the wave rim in order to surf faster toward the finish line”). Surfing is a real-life motor task that requires focused attention to external task-related stimuli (i.e., the incoming waves) besides a timely and accurate heading motor command to catch as many waves as possible to increase the distance covered while surfing. Therefore, we propose a novel stimulus-response paradigm: a virtual surfing task.

**FIGURE 2:**
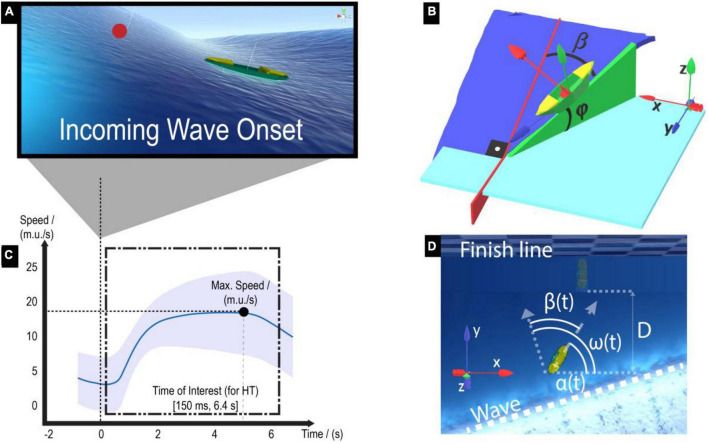
Virtual surfing environment and motor performance metrics for the Horizon Task (HT). **(A)** The boat could accelerate when an incoming wave reaches the boat. **(B)** The magnitude of the boat forward acceleration depends on the boat pitch angle (i.e., forward tilt φ). Note that the boat pitch angle is maximal when the alignment error β is minimal, i.e., when the participant aligns the boat pointing toward the wave direction. We depicted the x-direction of the global and boat local frames in red, the y-direction in blue, and the z-direction in green. **(C)** Catching a wave results in a speed increase. The trial-averaged speed profile along the time is plotted, taking all participants’ trials together. The mean and standard deviation of the speed across trials are represented by the solid blue line and the shaded area, respectively. The wave onset (time = 0 s) is marked with a vertical dotted line. **(D)** An exemplary incoming wave reaches the boat close to the finish line (checkerboard pattern). The dotted arrow represents the wave direction w.r.t. the x-direction of the global coordinate frame (angle ω). The dashed arrow represents the longitudinal boat direction w.r.t. the x-direction of the global coordinate frame (angle α). Performance metrics: the mean boat speed was computed as the mean boat horizontal speed (on the horizontal XY-global coordinates plane) within an interval of 150 ms until 6.4 s after incoming wave onset (namely, Time of Interest, ToI). The mean alignment error ($\bar{\beta }$), the distance surfed on the wave toward the finish line (**D**), and the joystick variability during the ToI were also computed.

Participants were asked to surf a boat on a wavy sea in a virtual environment using a joystick (Figure 1). The environment dynamics, which include the interaction of the boat with the water/waves, are rendered with the open-source software from the *Crest-OceanRenderer* community.^1^ The dynamics of the *Crest-OceanRenderer* software include gravity, buoyancy (i.e., floating force), and translation/rotational drag (i.e., fluid friction between the boat and water). The wave height and frequency of appearance are controlled across participants. The wave directions (angle ω in Figure 2D) are randomly selected from a pre-set of angles, with increments of 5°, within the range [60°, 120°] w.r.t. the x-direction of the global coordinates frame.

Participants could gain boat acceleration by surfing the waves. The magnitude of the boat forward acceleration *a_w_* (in *m*.*u*./*s*^2^, where *m.u.* stands for “maritime units”) depends on the boat pitch angle (angle φ in Figure 2B) according to the following equation:


(1) $$a_{w}\left(i\right)=\left\{ \begin{array}{cc} {\text{a}}_{w}\left(\text{i}-1\right)+{\text{c}}_{s}\cdot \varphi +0.1,\varphi \in \left[15^{{}^{{}^{\circ}}},90^{{}^{{}^{\circ}}}\right]\; & \\ {\text{a}}_{w}\left(\text{i}-1\right)<20\text{m}.u./{\text{s}}^{2}\; & \\ 20,\varphi \in \left[15^{{}^{{}^{\circ}}},90^{{}^{{}^{\circ}}}\right]\; & \\ {\text{a}}_{w}\left(\text{i}-1\right)\geq 20\text{m}.u./{\text{s}}^{2}\; & \\ {\text{a}}_{w}\left(\text{i}-1\right)-5\cdot {\text{c}}_{s}\cdot \varphi +2,\varphi \notin \left[15^{{}^{{}^{\circ}}},90^{{}^{{}^{\circ}}}\right]\; & \\ {\text{a}}_{w}\left(\text{i}-1\right)>0\text{m}.u./{\text{s}}^{2}\; & \\ 0,\varphi \notin \left[15^{{}^{{}^{\circ}}},90^{{}^{{}^{\circ}}}\right]\; & \\ {\text{a}}_{w}\left(\text{i}-1\right)\leq 0\text{m}.u./{\text{s}}^{2}\; & \end{array}\right.$$

where *i* represents each iteration step in the virtual environment and cs[0.17m.u./(s2⋅)°] is an empirically selected coefficient that increases the boat acceleration when the pitch angle (φ) is within [15°,90°], and decreases otherwise. Please note that the translational drag modeled within the *Crest-OceanRenderer* environment acts on top of Eq. (1) and reduces the boat’s acceleration due to friction forces.

The boat pitch angle φ is proportional to the wave slope and inversely proportional to the absolute alignment error β(*t*) = |ω(*t*) − α(*t*)|, where α(*t*) is the boat longitudinal direction angle w.r.t. the x-direction of the global coordinate frame and ω(*t*) is the wave direction angle w.r.t. x-direction of the global coordinate frame (Figures 2B,D). Thus, the smaller the alignment error was, the larger the boat pitch angle φ became, and following Eq. (1), the more the boat acceleration increased. Note that the boat only accelerates when the boat pitch angle is larger than 15°. The time when the pitch angle just reaches this 15° is denoted as the *incoming wave onset* (Figure 2A).

Participants could control the direction of the boat to reduce the alignment error by rotating the joystick about its vertical axis (γ_*z*_ in Figure 1). The angle of the joystick is mapped to a steering torque about the local boat yaw axis (i.e., the z component of the boat local frame, in green; Figure 2B) as:


(2) $$\left|\vec{\tau_{s}}\right|=\gamma_{z}\cdot {\text{c}}_{R}$$

where the value *c*_*R*_ = 0.27 *N*⋅*m*.*u*./° was empirically selected.

Altogether, to accelerate the virtual boat, participants needed to learn to reduce their alignment error, referred to as the **underlying task rule**.

### Motor Tasks and Experimental Design

**FIGURE 3:**
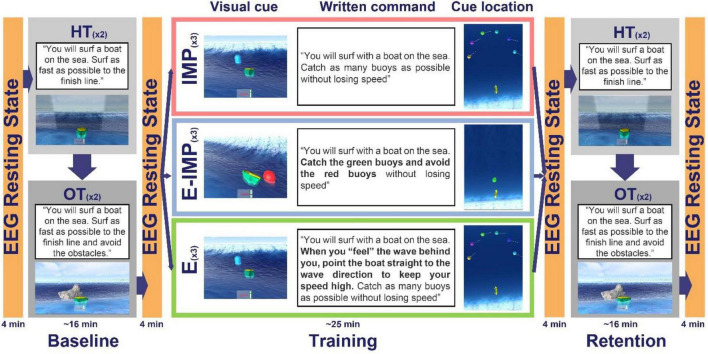
Study protocol overview. Different written explicit commands and visual cues were provided during each phase/task. During Baseline and Retention, participants completed the HT and the OT. During training, all participants were instructed to: “*Try to catch as many buoys as possible without losing speed*.” Depending on the Task Instruction Type, participants received different further instructions. Participants in the IMP group had no explicit information about the underlying task rule; they were just compelled to catch the buoys. Participants in the E group were compelled to catch the buoys while having the underlying task rule disclosed in written: “*When you feel the wave behind you, point the boat straight to the wave direction to keep your speed high*.” Participants in the E-IMP group were explicitly instructed to “*catch the green buoys*” and to “*avoid the red buoys*” to experience *caught* and *missed waves*, respectively.

The experiment consisted of three phases: Baseline, Training, and Retention (Figure 3). Participants were asked to complete two different test tasks during Baseline and Retention: a free surfing task (*Horizon Task*) and an obstacle avoidance surfing task (*Obstacle Task*). For a clearer understanding of the tasks, please see Supplementary Video 1.

The **Horizon Task** (HT) consisted of surfing the waves as fast as possible to a finish line (placed 2,500 m.u. ahead of the initial boat position on the global y-direction; Figure 2D) by catching as many waves as possible. Participants could see the boat current speed on a speedometer located next to the boat. The exact command provided to the participants before each HT task was: “*You will surf with a boat on the sea. Surf as fast as possible to the finish line.*”

During the **Obstacle Task** (OT), participants had to surf the boat as fast as possible to the finish line (as in the HT) while reacting immediately to avoid obstacles (stone blocks). To avoid the collision with the obstacles, participants had to be attentive at incoming wave onset (stimulus) and to either readily steer the boat at least 20° away from the wave direction when the obstacle appeared (reaction task) or align toward the wave direction when no obstacle appeared. The videogame interface presented the obstacles suddenly at incoming wave onset 10 m.u. from the boat current position along the wave direction (Figure 3), to limit planning processes. The obstacles were randomly presented on 50 % of incoming wave trials (*Obstacle* trials), while in 50 % of the incoming waves, no obstacles were presented (*Non-Obstacle* trials). Thus, the OT was used to assess whether participants would transfer the ability to steer the boat to catch waves, gained during training, into steering the boat and avoiding obstacles. The exact command provided to participants before each OT task was: “*You will surf with a boat on the sea. Surf as fast as possible to the finish line and avoid the obstacles.*”

Participants performed the HT twice during Baseline and Retention tests, followed by two OTs (4–5 min per task). Participants were exposed to ∼36 incoming waves (i.e., trials) per phase and task.

After a short break after Baseline (∼2 min), participants were randomly allocated to one of three different training groups (i.e., Task Instruction Type; Figure 3). Visual cues (i.e., floating buoys) appeared at incoming wave onset to create the training tasks. These visual cues provided information regarding the timing of an incoming wave onset.

The videogame interface also presented written instructions on the computer screen before each training task. Instructions were written using bold black 64 points Arial font over a white background and were displayed until the participant confirmed having read the full text and was ready to start the task. All participants, independently of the allocated Task Instruction Type group, were first provided with a written common command: “*You will surf with a boat on the sea. […]. Catch as many […] buoys as possible without losing speed.*” In two training groups, we supplemented this command to cue the required steering direction to gain speed (i.e., the underlying task rule) by either providing visual cues (E-IMP group) or explicit written commands (E group). The resulting training tasks (Figure 3), corresponding to three Task Instruction Types, differed in how explicitly the underlying task rule was provided.

#### Implicit Learning Without Instructions (IMP)

During training without instructions (IMP), single randomly coloured floating buoys appeared, at incoming wave onset, at one over six possible random locations over a semi-circumference of radius 25 m.u., centred 40 m.u. ahead of the boat in the wave direction, spanning from −90° to 90° w.r.t. the wave direction, where 0° corresponds to the wave direction. With this buoy distribution, we intended that participants would experience how the boat reacts (accelerates/deaccelerates) when aligning or misaligning the boat direction w.r.t. the wave direction. The exact command provided to participants in the IMP group before each training task on the computer screen was: “*You will surf with a boat on the sea. Catch as many buoys as possible without losing speed.*” No further explicit information was disclosed to participants about the underlying task rule to accelerate the boat.

#### Explicit Written Instructions (E)

During training with written instructions (E), single buoys were placed as in IMP. Before each training task, participants were provided with the following explicit command displayed on the computer screen: “*You will surf with a boat on the sea. When you ‘feel’ the wave behind you, point the boat straight to the wave direction to keep your speed high. Catch as many buoys as possible without losing speed.*” With this explicit written instruction, we intended to disclose the underlying task rule, uniquely to E participants. We checked before the Training phase that all participants understood what “pointing the boat straight to the wave direction” meant.

#### Explicit-Implicit Visual Cues (E-IMP)

During training with visual instructions (E-IMP), green “*Go*” buoys and red “*No-Go*” buoys were randomly presented on incoming wave trials and placed 10 m.u. ahead from the boat on the wave direction (50 % “*Go*” and 50 % “*No-Go*” buoys). The exact command provided via computer screen to participants in the E-IMP group before each training task was: “*You will surf with a boat on the sea. Catch the green buoys and avoid the red buoys without losing speed.*” Participants were explicitly instructed to “*catch the green buoys*” and to *“avoid the red buoys*” with the intention that only E-IMP participants would implicitly experience the boat heading angle needed to catch and miss waves, respectively. Importantly, green buoys were placed straight towards the wave direction so that participants would experience a speed increase (i.e., catch a wave). Conversely, red buoys were also placed straight in the wave direction, but as they were meant to be avoided, participants had to align the boat orthogonal to the wave direction to decrease their speed.

Each training task contained 36 incoming waves (trials) in which a floating buoy was presented. Participants performed the training task (∼ 7 min) three consecutive times.

After a short break (∼2 min), participants performed a Retention test with the same sequence of HT and OT tasks as during the Baseline. In addition, before, after, and between each phases, an EEG resting-state measurement (8 × 30 s Eyes Opened–Eyes Closed resting-state EEG) was performed. The total duration of the experiment was around 1.5 h.

The study’s primary outcomes are behavioural and neurophysiological analyses of the HT and the OT during the Baseline and Retention phases. These datasets can be found in a freely and openly available Zenodo-hosted repository (Penalver-Andres et al., 2021). The datasets of the Training phase and resting-state measurements are out of the scope of this publication.

### Data Processing and Statistical Analysis

#### Behavioural Data

##### Recording and Time of Interest

Kinematic data of the boat and joystick input were recorded at ∼50 Hz in Unity. Due to the uneven sampling rate, data were linearly interpolated at 50 Hz. To capture the participants’ performance while surfing the waves in the HT, we first inspected our dataset to determine the *Time of Interest* (ToI), i.e., the time interval within which we computed the performance metrics (Figure 2C). To determine the ToI, we first calculated the time to maximum boat speed on the horizontal XY-global coordinates plane (Figure 2D) across participants by taking Baseline and Retention HT trials together. The median and confidence interval (CI) of the time to maximum speed was 5.17 s (3.6–6.4 s) after incoming wave onset across all HT trials and participants. To account for the inter-subject variability, we selected a rather large conservative ToI by choosing the upper bound to be 6.4 s. We set the ToI lower bound at 150 ms after incoming wave onset to account for the participants’ visuomotor reaction times (Laming, 1968). In the OT, the ToI was extended from 0 to 7 s after incoming wave onset to capture the entire obstacle avoidance process, i.e., from obstacle detection to surfing around the obstacle.

##### Performance Metrics

Given the novelty of our task—as opposed to classical stimulus-response paradigms—using conventional performance variables, such as detection or reaction time was not possible (Haegens et al., 2011; Payne et al., 2013; Schneider et al., 2019). Our novel, real life-based paradigm requested to find behavioural metrics that would quantify the potential cognitive-attentional advantages of different training strategies. As such, we selected meaningful task-specific performance metrics based on those previously employed to assess motor learning in literature (i.e., velocity, error, and variability; Basalp et al., 2021). Employing the boat velocity, steering error, and joystick variability (for the HT) and additionally the detection-reaction metrics (for the OT) provides a good overview of the participants’ task performance before and after training, which we expected to be supported by attentional processes, especially early during training (Poulter et al., 2005; D’Innocenzo et al., 2016; Cardoso et al., 2019).

In the HT, participants were asked to “*surf as fast as possible to the finish line.*” Thus, the **mean boat speed** [*mean*(*v*)], was computed as the mean boat speed on the horizontal XY-global coordinate plane ($v=\sqrt{v_{x}^{2}+v_{y}^{2}}$, being *v*_*x*_ and *v*_*y*_ the x and y components of the boat velocity, respectively) within the ToI. The boat speed data were low-pass filtered with a second-order Butterworth filter (cut-off frequency of 5 Hz) to filter out the medium-frequency components of the speed signal resulting from the participants’ variable joystick commands. The filtered speed signal was employed to calculate the upper bound of the ToI (i.e., the time of maximum/peak speed).

Since the wave direction varied between waves (within the [−30°, 30°] range), and therefore, aligning the boat to the wave direction did not always result in advancing in a straight line to the finish line, we also calculated how much the boat advanced in the y-direction of the global coordinate frame toward the finish line during the ToI (**distance surfed toward the finish line**; $D=\left|p_{y}^{t=6.4s}-p_{y}^{t=0.15s}\right|$, where *p*_*y*_ is the boat position along the y-direction of the global coordinate frame, orthogonal to the finish line, at *t* = 6.4*s* and *t* = 0.15*s* from incoming wave onset; Figure 2D).

The **boat alignment error** was calculated as the mean absolute alignment error β during the ToI (Figure 2D). Alignment errors close to zero reflect the participant’s ability to perform the underlying task rule (“*point the boat straight to the wave direction to keep your speed high*”).

The **joystick variability** was computed as the standard deviation of the joystick rotation about its vertical axis (γ_*z*_). This metric quantifies the variability of participants’ steering commands while surfing the waves.

All metrics were computed by pooling *Hit* and *Missed Wave* trials together. A trial was considered as *Hit Wave* trial when the participant caught an incoming wave, i.e., when the mean boat speed during the ToI increased w.r.t. to the instantaneous boat speed at the start of the ToI (*v*_0_ = *v*^*t* = 0.15*s*^). Otherwise, the trial was considered a *Missed Wave* trial. A slight decay of the mean boat speed (2 % of *v*_0_) was allowed to account for the deaccelerating effects of the drag on the boat.

The same kinematic metrics were computed during the OT for *Obstacle* trials. For the OT, two additional metrics were computed to characterize the ability of participants to avoid collisions: success rate and obstacle avoidance inability. In the OT, four possible trial outcomes are possible: (1) true positive, i.e., the participant misaligns the boat to avoid obstacle collision; (2) true negative, i.e., the participant keeps surfing when no obstacle appears; (3) false positive, i.e., the participant misaligns the boat although no obstacle appears; and (4) false negative, i.e., the participant does not misalign the boat when an obstacle appears. The **success rate**, a typical stimulus-response paradigm metric was calculated as the ratio of total true positives and true negatives divided by the total number of trials. This metric characterizes the correctness of participants’ actions, i.e., whether participants misaligned the boat to avoid the obstacle and did not misalign the boat when there was no obstacle. To further characterize participants’ **obstacle avoidance inability**, the ratio of total false negatives divided by the total number of trials was computed.

##### Statistical Analysis

**FIGURE 4:**
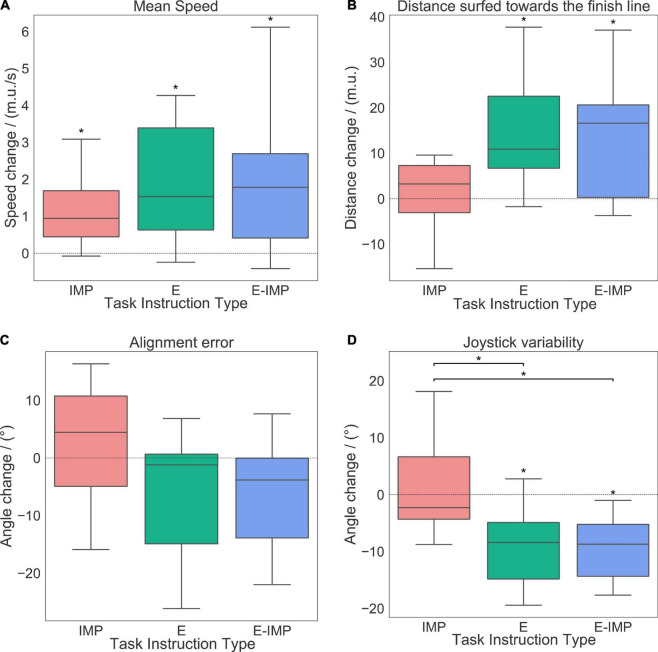
Changes in performance metrics [mean speed **(A)**, distance surfed towards the finish line **(B)**, alignment error **(C)** and joystick variability **(D)**] pre-post training for the HT, per metric and group: whiskers show the data ranging 1.5 times inter-quartile range above the upper or below lower quartiles, boxed horizontal solid lines represent the median values and box vertical boundaries represent the inter-quartile range of the metric pre-post training changes. Positive values represent a pre-post training increase. Between-group (horizontal connectors) and within-group (above boxplot) significant differences (*p <* 0.05) are marked with *.

The average of each kinematic performance metric across all trials (for the HT) and *Obstacle* trials (for the OT) during each test phase (i.e., Baseline and Retention) was computed per participant. The success rate and obstacle avoidance inability metrics (for the OT) were calculated with both *Obstacle* and *Non-Obstacle* trials. The pre-post training change (i.e., from Baseline to Retention) in the performance metrics was computed for each participant (Figure 4 and Table 1).

**TABLE 1:** Pre-post training behavioural changes within-group and between-group comparisons.

	Pre-post training changes within group			Between group tests
	IMP	E	E-IMP	*KW (*p*-value)*
	**HT Task**			

Mean speed	0.94 (0.37–1.91) m.u./s *10.0 (****0.02***)*	1.53 (0.47–3.53) m.u./s *4.0 (****0.006***)**	1.78 (0.19–2.79) m.u./s *6.0 (****0.009***)**	*0.91 (0.61)*
Distance surfed toward finish line	3.24 (−3.24–8.21) m.u. *25.0 (0.27)*	10.91 (3.87–22.92) m.u. *6.0 (****0.01***)*	16.63 (−0.77–23.82) m.u. *9.0 (****0.02***)*	*3.06 (0.22)*
Alignment error	4.46 (−5.47–11.60)^°^ *30.0 (0.48)*	−1.21 (−15.57–0.85)^°^ *19.0 (0.12)*	−3.81 (−15.36–0.02)^°^ *14.0 (0.05)^∙^*	*3.81 (0.15)*
An error in the conversion from LaTeX to XML has occurred here. gray!50				
An error in the conversion from LaTeX to XML has occurred here. gray!50				
Joystick variance	−2.27 (−4.74–6.80)^°^ *38.0 *(0.94)**	−8.4 (−16.31–−4.31)^°^ *3.0 (****0.005***)**	−8.69 (−5.03–−0.09)^°^ *0.0 (****0.002***)**	*10.82 (* ***0.004*** )**

	**OT Task**			

Mean speed	1.41 (−0.19–4.22) m.u./s *16 (0.07)^∙^*	*1.11 (−1.84–2.64) m.u./s* 32 *(0.59)*	1.01 (0.57–2.79) m.u./s *11 (****0.02***)*	*0.66 (0.72)*
Distance surfed toward finish line	15.92 (−3.43–32.93) m.u. *14 (0.05)^∙^*	6.51 (−6.89–37.55) m.u. *62 (0.36)*	11.55 (8.27–22.07) m.u. *3 (****0.005***)**	*0.62 (0.73)*
Alignment error	−5.07 (−10.82–4.04)^°^ *22 (0.18)*	−7.00 (−9.73–2.86)^°^ *20 (0.14)*	−10.50 (−12.55–−2.78)^°^ *3 (****0.005***)**	*1.71 (0.42)*
Joystick variance	−1.31 (−5.52–6.64)^°^ *36 (0.81)*	−1.42 (−3.94–2.56)^°^ *31 (0.53)*	−6.17 (−12.08–2.69)^°^ *20 (0.14)*	*2.81 (0.25)*
An error in the conversion from LaTeX to XML has occurred here. gray!50				
An error in the conversion from LaTeX to XML has occurred here. gray!50				
Success rate	−13.14 (13.50) % *11 (****0.02***)*	4.74 (19.88) % *45 (0.67)*	3.27 (10.42) % *51 (0.38)*	^†^*4.74* (***0.02***)*
Obstacle avoidance inability	10.15 (−1.22–16.74) % *62 (0.07)^∙^*	−10.66 (−21.28–11.87) % *25 (0.30)*	−7.52 (−17.47–4.39) % *21 (0.17)*	*2.00 (0.37)*

**Descriptive statistics:** Mean (standard deviation) or median (25 % quantile–75 % quantile) range are reported. **Within-group comparisons**: Retention-Baseline differences that reached statistical significance are marked with bold values. Wilcoxon signed-rank p-values are reported. **Between-group comparisons**: Metrics with Retention-Baseline differences that show an effect of Task Instruction Type are shaded in gray. Kruskal-Wallis H-Test statistic (^†^One-way ANOVA χ-squared) is reported Test statistic values are marked in italic with p-values written in brackets. (^∙^p < 0.1, *p ≤ 0.05, **p ≤ 0.01). “°” stands for degrees.

Differences in Baseline performance across groups (factor Task Instruction Type: E, IMP, E-IMP) were tested for each performance metric (Kruskal-Wallis H-Test, KW-H, *p > 0.1*). Wilcoxon signed-rank test (WSR) was used to test whether each group significantly changed a performance metric pre-post training. Assumptions for parametric testing were checked using normality tests (Kolmogorov-Smirnov, *p < 0.05*) and Homogeneity tests (Levene’s test, *p > 0.05*).

To test between-group (Task Instruction Type) differences in performance change after training, Retention minus Baseline datasets were compared using parametric or non-parametric tests, when applicable. For parametric testing, One-way ANOVA was used followed by *post-hoc t*-tests with Tukey-Kramer multiple-comparison correction. Otherwise, Kruskal-Wallis H-Test was used combined with *post-hoc* Bonferroni-Holmes corrected Mann-Whitney *U* tests (MW-U). The significance level was set to *α < 0.05* for all tests.

To report descriptive statistics, we employed the mean and standard deviation (SD) when data were normally distributed and the median and inter-quantile range (IQR) otherwise. To report effect size, *r*_*Cohen^’ s  d*_ is provided when significant contrasts were detected. Behavioural data processing and analysis of the HT were performed using Python 3.7.1 and libraries Matplotlib, NumPy 1.15.4, pandas 0.23.4, statsmodels 0.9.0, and SciPy 1.1.0. Matlab (MathWorks, United States) was used to process and analyse the data of the OT.

#### Electrophysiological Data

##### Preprocessing

EEG data were sampled at 1,000 Hz and preprocessed offline following the standard procedure implemented in the Matlab-based toolbox Automagic (Pedroni et al., 2019). We included 186 electrodes with a high signal-to-noise ratio in the preprocessing, excluding electrodes at array boundaries heavily confounded with muscle artifacts in the neck, maxillary, mandibular, and eyebrow areas (Supplementary Figure 1). Preprocessing consisted of line noise removal (50 Hz), average reference and band-pass (between 0.1 and 40 Hz) filtering using the EEGLAB function *eeg_filtnew* (Delorme and Makeig, 2004), and detection of bad channels using the toolbox PREP (Bigdely-Shamlo et al., 2015) called from Automagic. In addition, ocular artifacts (i.e., eye blinks and movements) were regressed out from the continuous signal using the signal of eye-neighbouring electrodes, which were marked as eye oculography (EOG) channels (for more information about the algorithm, please refer to Parra et al., 2005). Finally, previously detected bad channels were interpolated using spherical interpolation with EEGLAB function *eeg_interp*.

##### Electrophysiological Metrics

Electroencephalography single-trial epochs ranging from 1,000 ms pre-stimulus (i.e., incoming wave) onset to 1,000 ms post-stimulus onset were extracted from the preprocessed data. At Baseline and Retention, an average of 43 ± 10 (for the HT) and 69 ± 19 (for the OT) single-trial epochs were extracted for each participant (with no statistical differences found across groups). Temporal-Spectral Evolution (TSE) (Thut et al., 2006) of the alpha-band (7–15 Hz) signal reflecting alpha wave strength was extracted for the time window [−1 s, +1 s] from incoming wave onset across all electrodes for each single-trial epoch. For this, the EEG single-trial epoched data were band-pass filtered (7–15 Hz), rectified and low-pass filtered using a cut-off boundary of half of the low-cut frequency. This procedure provides a smoothed electric scalp amplitude (*μV*) map for the signal contained in a specific frequency band which is a positive definite across time points, informing about the topographic distribution of alpha-wave strength. The single-trial TSE was averaged per participant (belonging to a specific Task Instruction Type), Trial Type (Hit/Missed Wave in the HT; Obstacle/Non-Obstacle in the OT), and Phase (Baseline/Retention) using EEGLab Toolbox (Delorme and Makeig, 2004).

##### Statistical Analysis

Statistical analyses were conducted using Ragu for Matlab (Koenig et al., 2011). Differences in the alpha-wave topographic distribution for the time window [−1 s, +1 s] relative to incoming wave onset were tested with a mixed-measures topographic ANOVA (TANOVA; Koenig et al., 2011; Habermann et al., 2018) with the between-subject factor Task Instruction Type (E, IMP, E-IMP) and the within-subject factors Phase (Baseline, Retention) and Trial Type (Hit/Missed Wave in the HT; Obstacle/Non-Obstacle in the OT). TANOVA is based on randomization statistics (here, 5,000 permutations per data point were used) and tests for significant differences in topographic distribution (*p < 0.05* for all electrophysiology-related statistics), indicating if different sources (i.e., brain networks) were active between factor levels. TANOVAs were initially computed for each time point and thresholded at a 5 % *p*-value. To control for multiple testing across time points, the duration of continuous periods of sub-threshold *p*-values corresponding to a 5 % false-positive rate were estimated (Koenig and Melie-García, 2010). Only sub-threshold periods with durations larger than this critical duration (279 ms for HT and 316 ms for OT) were further analysed. For the subsequent analyses, the data were averaged within these windows and single TANOVAs on these averages were computed and reported.

In the case of a 3-way Phase × Trial Type × Task Instruction Type interaction effect, we conducted *post-hoc* one-factor pairwise TANOVA comparisons of Trial Type contrast maps in the averaged time window showing significant interaction effects. The Trial Type contrast maps were defined as the subtraction of the alpha-wave topographic distributions of different Trial Types in each task—e.g., Hit Waves minus Missed Waves (for the HT). These contrast maps characterize the learning of the task as the neural distance between the two bifurcation sides (i.e., Hit/Missed Wave or Obstacle/Non-Obstacle, respectively, for the HT and the OT) (Chadick and Gazzaley, 2011). Thus, the alpha-wave topographic distribution contrasts reflect the differentially active parts of attentional networks responsible for participant’s Trial Type discrimination skills (namely, visuo-attentional spatial acuity; Montagna et al., 2009), which we expected to be modulated as a function of Task Instruction Type after training.

**FIGURE 5:**
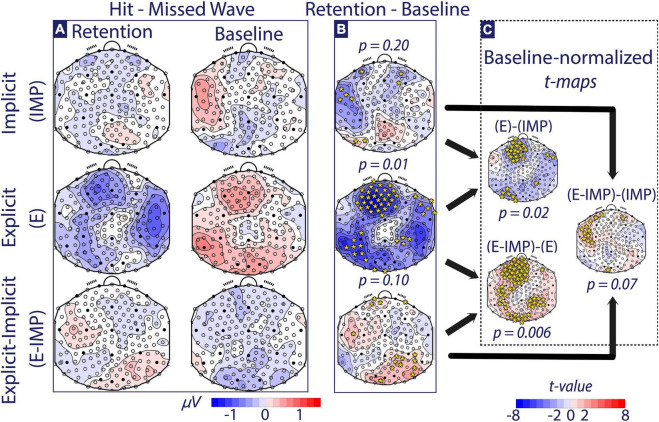
Alpha-wave Trial Type contrast maps (Hit-Missed waves). **(A)** The alpha-wave Trial Type contrast maps at Baseline and Retention are depicted for each Task Instruction Type (micro-Volts shown). **(B)** The Retention–Baseline contrast is depicted for each Task Instruction Type (micro-Volts shown). **(C)** The Baseline-normalized between-group *t*-maps are depicted (*t*-values shown). Colour coding for panels **(A,B)** is depicted at the bottom of the figure in micro-Volts. All scalp maps present the electrode positions overlaid. Electrodes marked with full circles correspond to the 10–20 EEG electrode convention. In panels **(B,C)**, time-averaged TANOVA *p*-values are shown for each contrast. Additionally, electrodes with |*t*-value| *> 2.36* (i.e., significance threshold) are depicted with in yellow *.

**FIGURE 6:**
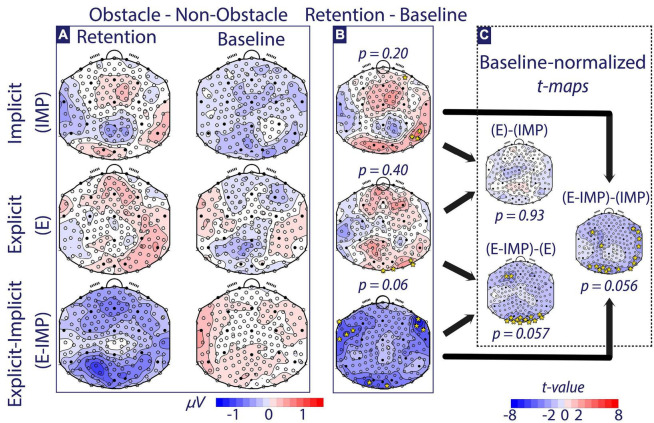
Alpha-wave Trial Type contrast maps (Obstacle–Non-Obstacle Waves). **(A)** The alpha-wave Trial Type contrast maps at Baseline and Retention are depicted for each Task Instruction Type (micro-Volts shown). **(B)** The Retention–Baseline contrast is depicted for each Task Instruction Type (micro-Volts shown). **(C)** The Baseline-normalized between-group *t*-maps are depicted (*t*-values shown). Colour coding for panels **(A,B)** is depicted at the bottom of the figure in micro-Volts. All scalp maps present the electrode positions overlaid. Electrodes marked with full circles correspond to the 10–20 EEG electrode convention. In panels **(B,C)**, time-averaged TANOVA *p*-values are shown for each contrast. Additionally, electrodes with |*t*-value| *>* 2.36 (i.e., significance threshold) are depicted with yellow *.

We focused on three specific contrasts of interest. First, to test whether groups presented different attentional networks during Baseline, we conducted *post-hoc* one-factor pairwise TANOVA comparisons across the Baseline alpha-wave Trial Type contrasts between Task Instruction Type. Second, to detect if attentional brain networks significantly changed pre-post training, we compared Retention and Baseline alpha-wave Trial Type contrast maps within each Task Instruction Type group (panel B in Figures 5, 6). Finally, to investigate differences in attentional networks linked to training with different Task Instruction Type, alpha-wave Trial Type contrast maps were compared via *post-hoc* one-factor pairwise TANOVA comparisons between Task Instruction Type on baseline-normalized (i.e., Retention–Baseline) contrast maps (panel C in Figures 5, 6). To visualize significant TANOVA effects, we computed electrode-wise *t*-tests of the significant contrasts and displayed this as topographic maps, highlighting electrodes that reached a threshold that would correspond to an uncorrected *p*-value of 5 % (*t* > 2.36; *t*-maps; Koenig et al., 2011) in Figures 5B,C, 6B,C. Note, however, that the statistical hypothesis testing was not based on these *t*-maps but solely on the TANOVAs.

## Results

### Behavioural Data

#### Effect of Task Instruction Type on Motor Performance Changes in Horizon Task

Participants’ motor performance (i.e., mean speed, distance surfed toward the finish line, alignment error, and joystick variability) did not differ across groups at Baseline (factor: Task Instruction Type; Kruskal-Wallis H-test, *p > 0.1*).

All groups significantly increased their **mean speed** on the waves after training (Figure 4A and Table 1; WSR, *p ≤ 0.05*). No difference between groups was found in the mean speed pre-post training change (Table 1).

Participants in the E and E-IMP groups significantly improved the **distance surfed toward the finish line** on the wave after training (Figure 4B and Table 1; WSR, *p < 0.05*). However, we did not find a statistically significant effect of Task Instruction Type on the pre-post training change of distance surfed toward the finish line (Table 1).

Only participants in the E-IMP group tended to reduce their **alignment error** after training (Figure 4C and Table 1; WSR, *p = 0.05*). However, we did not find a statistically significant effect of Task Instruction Type on the alignment error change pre-post training (Table 1).

Finally, participants in the E and E-IMP groups reduced their **joystick variability** significantly after training (Figure 4D and Table 1; WSR, *p < 0.01*). We found a significant effect of Task Instruction Type on the joystick variability change pre-post training (Figure 4D and Table 1; Kruskal-Wallis H-test, χ^2^ = 10.82, *p = 0.004*). In particular, the IMP group reduced the joystick variability significantly less than the E-IMP (Mann-Whitney *U* test, *U = 19*, *p = 0.003*, *r*_*Cohen*′*sd*_ = *1.46*) and E groups (Mann-Whitney *U* test, *U = 27*, *p = 0.01*, *r*_*Cohen*′*sd*_ = *1.04*).

#### Effect of Task Instruction Type on Motor Performance Changes in Obstacle Task

Participants’ Baseline performance metrics did not differ significantly across Task Instruction Type in the OT (Kruskal-Wallis H-test or one-way ANOVA, *p > 0.1*).

Participants in the E-IMP group increased the **mean speed** on the wave pre-post training (Table 1; WSR, *p = 0.02*). We only found a statistical trend for an increase in the mean speed in the IMP group (Table 1; WSR, *p = 0.07*). The IMP and E-IMP groups also increased the **distance surfed toward the finish line** (Table 1; WSR, *p ≤ 0.05*) from Baseline to Retention in the OT. Only participants in the E-IMP group significantly reduced the **alignment error** pre-post training (Table 1; WSR, *p = 0.005*). However, no significant effect of Task Instruction Type was found in any of the kinematic metrics (Table 1).

Conversely, participants in the IMP group significantly decreased their **success rate** pre-post training (Table 1; WSR, *p = 0.02*). We found a main effect of Task Instruction Type on the success rate **[**Table 1; one-way ANOVA, *F(2, 33) = 4.74*, *p = 0.02*]. *Post-hoc* tests revealed that the participants in the E (Tukey-Kramer *t*-test, *t = 2.56*, *p = 0.02*, *r*_*Cohen*′*sd*_ = *1.63*) and the E-IMP (Tukey-Kramer *t*-test, *t = 4.90*, *p = 0.04*, *r*_*Cohen*′*sd*_ = *1.50*) groups increased their success rate significantly more than participants in the IMP group.

In the same line, participants in the IMP group increased (without reaching statistical significance) their **obstacle avoidance inability** pre-post training (Table 1; WSR, *p = 0.07*). No statistically significant differences were found between groups (Table 1).

### Electrophysiological Data

#### Effect of Task Instruction Type on Attentional Networks During Horizon Task

We investigated differences in alpha-wave topographic distribution using a mixed-measures Phase × Trial Type × Task Instruction Type TANOVA. In the interval comprising −130 ms pre-stimulus to 170 ms post-stimulus (i.e., incoming wave onset), we found a continuous period with a significant (*p < 0.05*) Phase × Trial Type × Task Instruction Type interaction effect. The duration of this effect was larger than 95 % of the duration of such effects that one would expect under the null hypothesis (Koenig and Melie-García, 2010), making it unlikely that the finding can be explained by multiple testing. The TANOVA test in the averaged [−130 ms, 170 ms] period rejected the null hypothesis (*p* < 0.001). Thus, we found a significantly different modulation of attentional networks observed post-training across Task Instruction Type depending on the Trial Type. Thus, the remainder of the *post-hoc* pairwise TANOVA comparisons comprises only the averaged [−130 ms, 170 ms] time interval.

At Baseline (Figure 5A, right column), these *post-hoc* tests only resulted in one significant difference between E and E-IMP groups, based on one-factor pairwise TANOVA comparisons of Trial Type contrast maps across Task Instruction Type (*p = 0.04*; less alpha-wave strength in E-IMP compared to E group over frontal and parieto-occipital areas). At Retention, the corresponding *post-hoc* one-factor pairwise TANOVA comparisons of Trial Type contrast maps also differed between E and E-IMP groups; however, the effect pointed in the opposite direction (*p = 0.03*; more alpha-wave strength in E-IMP compared to E group over frontal and parieto-occipital).

Further, we tested if attentional brain networks significantly changed pre-post training within each group (Figure 5B). Participants allocated in the E group showed a significant change in attentional brain networks pre-post training (*p = 0.01*; less alpha-wave strength at frontal, right parietal-temporal, and left parieto-occipital sites during Retention as compared to Baseline). Similarly, participants in the E-IMP group showed pre-post training changes in observed attentional networks, yet those did not reach the significance threshold (*p = 0.10*; more alpha-wave strength at frontal, occipital and right parietal sites during Retention compared to Baseline). Conversely, participants allocated in the IMP group showed no significant differences in alpha-wave Trial Type contrast maps when comparing pre-post training.

Finally, to investigate if Task Instruction Type modulated the observed pre-post training changes in attentional brain networks, alpha-wave Trial Type contrasts were compared between Task Instruction Type based on baseline-normalized (i.e., Retention–Baseline) contrast maps (Figure 5C). The three resulting contrasts between groups were all significant or at least approached statistical significance (*p* < 0.05). Group E-IMP showed a stronger pre-post training increase in parieto-occipital and frontal alpha wave strength than the E group (*p = 0.006*; more alpha-wave strength at frontal, occipital and right parietal sites post-training). Compared to the IMP group, the E-IMP group tended to show a stronger pre-post training increase in parieto-occipital alpha wave strength than the IMP group (*p = 0.07*; occipital sites). Finally, participants in the E group showed a stronger pre-post training decrease in occipital and frontal alpha wave strength than the IMP group (*p = 0.02*).

#### Effect of Task Instruction Type on Attentional Networks During Obstacle Task

Identical analyses were performed on the OT dataset. We investigated differences in alpha-wave topographic distribution using a mixed-measures Phase × Trial Type (i.e., Obstacle–Non-Obstacle) × Task Instruction Type TANOVA. In the time interval the interval comprising −50 ms pre-stimulus to 350 ms post-stimulus (i.e., incoming wave onset), we found a significant (*p < 0.05*) Phase × Trial Type × Task Instruction Type interaction effect. The TANOVA test in the averaged [−50 ms, 350 ms] period rejected the null hypothesis (*p = 0.03*). Thus, there was a significantly different modulation of attentional networks observed pre-post training across Task Instruction Type depending on the Trial Type. Noteworthy, this latter this latter interval overlapped in time with the previously reported HT interaction effect. As the interval of interest was more extensive than 95 % of the durations of significance expected under the null hypothesis, we can confidently state that our results are robust against multiple comparisons-related biases. The remainder of the *post-hoc* pairwise TANOVA comparisons comprises only the averaged [−50 ms, 350 ms] time interval.

At Baseline (Figure 6A, right column), *post-hoc* one-factor pairwise TANOVA comparisons among Trial Type contrast maps did not significantly differ across Task Instruction Type (*p ≥ 0.053*). Further, we tested if attentional brain networks significantly changed pre-post training within each group (Figure 6B). Participants allocated in the IMP and E groups showed no significant difference in alpha-wave Trial Type contrast maps pre-post training. Participants allocated in the E-IMP group tended to change the pre-post training attentional brain networks (*p = 0.06*; less alpha-wave strength at bilateral fronto-temporal and parieto-occipital electrodes).

Finally, to investigate if task instructions modulate the attentional brain networks observed, alpha-wave Trial Type contrast maps were compared between Task Instruction Type based on baseline-normalized (i.e., Retention–Baseline) contrast maps (Figure 6C). Participants in the E-IMP group showed nearly significantly (*p ≤ 0.057*) different pre-post training changes in attentional networks when compared to IMP and E groups (Figure 6C shows significant electrode comparisons and corresponding *post-hoc* comparison test): Group E-IMP showed a stronger pre-post training decrease in parieto-occipital and frontal alpha wave strength than the IMP and the E groups.

## Discussion

In this study, we investigated how providing different (visually cued or written) task instructions during training affects participants’ attention while performing a virtual surfing motor task. We used two motor tasks in a virtual environment: free surfing (HT) and obstacle avoidance surfing (OT) tasks.

### Providing Instructions About the Underlying Task Rule Improves Motor Performance in the Free Surfing Task

In the free surfing task HT, we found that participants trained with explicit written instructions (E) and with explicit visual cues (E-IMP) significantly improved their surfing performance pre-post training. In particular, participants in the E and E-IMP groups decreased the joystick variability from Baseline to Retention to a significantly greater extent than the IMP group. However, the within-group pre-post training changes found mainly in E and E-IMP groups in the other kinematic variables (distance surfed on the waves, alignment error, and mean speed) were not found to be significantly different across groups.

Along with the improvements observed in all groups, we found that training with explicit instructions resulted in less variability of the motor command to control the boat direction (i.e., participants in the E and E-IMP group significantly reduced the joystick variability pre-post training) to a greater extent than the IMP group. Small motor variability has been consistently associated with the expert stage of motor learning, while more variable movements are characteristic of the early stages of motor learning (Cohen and Sternad, 2009; Dhawale et al., 2019). Thus, the higher joystick variability observed in participants in the IMP group, compared to other groups, could reflect that they were still in the relatively early stages of the learning process during Retention, probably still embarked on the search for an underlying task rule to skilfully achieve the task (Albinet and Fezzani, 2003).

The enhanced performance observed in the E and E-IMP groups after training is consistent with previous literature showing that visual cues and explicit commands support motor learning (Hardy et al., 2001; Albinet and Fezzani, 2003; Janelle et al., 2003; Wolpert et al., 2011; D’Innocenzo et al., 2016). The pre-post training motor performance improvement observed in the E and E-IMP groups in the free surfing task may result from enforced top-down processes that draw participants’ attention toward task-relevant stimuli. For example, in the study of Poulter et al. (2005), participants who were instructed to pay attention to specific postural aspects of a soccer penalty kicker enhanced their accuracy in predicting the shot direction compared to uninstructed participants. Poulter et al. (2005) suggested that explicit instructions support a quicker transition between the early cognitive and the later associative and autonomous stages of motor learning.

Further, training without knowledge of the underlying rules is linked to lesser declarative knowledge generation (i.e., an understanding that can be verbalized, e.g., “aligning the boat to the wave direction propels the boat faster”), compared to training with explicit written (Koedijker et al., 2007, 2011) or visual (D’Innocenzo et al., 2016) instructions. Therefore, knowledge of the task acquired via (visually cued and written) task instructions may support participants’ attentional focus on task-relevant stimuli to generate effective and controlled responses (Poulter et al., 2005), potentially accelerating motor learning.

The command provided before training to all training groups instructed participants to catch (or avoid) as many buoys as possible (primary goal: accuracy) without losing speed (secondary goal: speed). Thus, the specific order of the commands equally enforced accuracy over speed across all training groups, potentially facilitating learning, as it has been shown that getting quicker in the movement does not imply getting better at a task (Batmaz et al., 2016). Therefore, we do not expect speed-accuracy trade-off effects to have differently impacted our training groups. The HT in baseline and retention tests did not enforce accuracy (no buoys were added), while in the OT the instructions were reversed, compared to the training tasks, i.e., participants were instructed to surf as fast as possible and avoid obstacles. We chose to enforce speed over accuracy in both test tasks to resemble a regatta scenario (i.e., the time to reach a finish line is the typical qualifying criteria) and to evaluate whether participants complied with the underlying task rule. Although the initial skill level of the participants might also influence our results (Marchal-Crespo et al., 2009, 2015), we did not find differences in the performance metrics between training conditions at baseline, and therefore, our findings are unlikely explained by different skill levels across groups.

### Visual Cueing and Written Instructions Engage Different Learning-Related Attentional Networks

To investigate whether task instructions affected attentional networks, we analysed the average global alpha-band topographical distribution within the EEG recordings. Decreased alpha-band power has been traditionally linked to enhanced neuronal processing of stimuli in primary sensory and association cortices—e.g., visual stimuli and occipital alpha-band activity (Thut et al., 2006)—while increased alpha-band power seems to help to suppress task-irrelevant stimuli (Haegens et al., 2011; Klimesch, 2011, 2012; Payne et al., 2013). Although the role of the amplitude of alpha-band oscillations is yet undeciphered, i.e., whether these oscillations represent irrelevant-stimuli suppression or relevant-stimuli enhancement, there is enough evidence that large-scale alpha-band networks are the backbone of top-down modulated attention (Palva and Palva, 2007; Britz et al., 2010; Jann et al., 2010; Zanto et al., 2011; Klimesch, 2012). To avoid controversial interpretations, we focused on global topographic changes, which can be directly interpreted as the engagement of distinct brain networks (e.g., Lehmann and Skrandies, 1980), instead of local alpha-band modulations. Nevertheless, we chose to report regional activation patterns for descriptive purposes.

In support of our behavioural data, we found that the task instructions modulated the engagement of attentional networks during training. The E and E-IMP groups changed the average topography of the alpha-band wave amplitude pre-post training (although in the E-IMP group did not reach statistical significance).

In the E-IMP group, we found increased alpha-band amplitude over the frontal and parieto-occipital areas pre-post training. The best explanation for this distribution is that those areas correspond to the frontoparietal network of visual attention (FPN; Parr and Friston, 2017). It is known that the prefrontal cortex, the premotor cortex, the frontal eye field, the intraparietal sulcus, and the posterior visual areas are part of this network. Several studies (Buschman and Miller, 2007; Zanto et al., 2010; Chadick and Gazzaley, 2011; van Driel et al., 2017; Nydam et al., 2018) have shown that the prefrontal cortex communicates via long-range alpha-band wave coupling with other FPN areas. Two functions are associated with such long-range coupling: (1) filtering task-related visual stimuli (Bressler et al., 2008; Del Percio et al., 2009; Chadick and Gazzaley, 2011; Haegens et al., 2011; Payne et al., 2013; Horschig et al., 2014; Schneider et al., 2019), and (2) controlling whether saccadic eye movements become exploratory (to detect new task-relevant stimuli) or exploitatory (toward task-relevant stimuli) (Astafiev et al., 2003; Chadick and Gazzaley, 2011; Brosnan et al., 2020; Gaillard et al., 2020). Under this framework, we can conclude that participants in the E-IMP group could have relied on FPN-mediated processes during training (Bressler et al., 2008; Payne et al., 2013; Schneider et al., 2019), enhancing their performance after training (Astafiev et al., 2003; Brosnan et al., 2020).

In the E group, we observed a pre-post training decrease of the alpha-band power predominantly over the right temporal and central-frontal areas. Temporal brain areas have been linked to working memory processes (Palva et al., 2011; Buszard et al., 2016) and central-frontal areas to motor planning processes (Albares et al., 2014). These areas are known to operate coupled via alpha-band waves (Buszard et al., 2016). Furthermore, learning a motor task with explicit instructions has been related to higher working memory demands than implicit learning (i.e., without explicit instructions). In several studies (Zhu et al., 2011a, b; Buszard et al., 2016; van Duijn et al., 2019), higher working memory demands linked to explicit instructions were reflected in an increased co-activation of the motor and verbal-analytical areas compared to implicit learning, in the alpha-band spectrum. In these studies, no behavioural differences were found between explicit and implicit motor learning. Additionally, alpha-band suppression (higher neural activation) has been linked to working memory-related networks (Klimesch, 1996; Jann et al., 2010). Participants performing a sustained visual attention task showed alpha-band power suppression over parieto-occipital areas when participants are informed about the task rule that encodes the presentation of visual stimuli (van Driel et al., 2012). The authors link this finding to a verbal working memory-supported “refocusing” of attention when competing stimuli are presented (van Driel et al., 2012, 2017), similarly to the floating buoys presented to E participants during training. Therefore, under this evidence, the most feasible explanation is that the processing of written instructions required joint functioning of motor, visual, or verbal-analytical areas involved in supporting working memory of the task rule during motor learning.

Participants in the IMP group showed no consistent changes in the alpha-band sources pre-post training. Thus, their attentional processes did not differ consistently from that of the Baseline, possibly impacting their ability to discriminate and select the correct movement patterns needed to catch or miss a wave. Two interpretations are possible. First, participants in the IMP group might have deployed several training strategies involving different neural processes, which yield no consistent significant pre-post training changes. Inconsistent strategy selection could result from IMP participants having no other means to learn the task but exploring the environment to infer a rule. Alternatively, participants in the IMP group may have needed a longer time to master the motor task and, thus, involve the required attentional resources. A slower learning rate could explain why we did not find consistent neurophysiological pre-post training changes in the IMP group. As opposed to explicit learning, slower learning rates in implicit learning have been consistently reported in the literature, especially during the early stages of motor learning (Janelle et al., 2003; Poulter et al., 2005).

Comparisons between groups showed that training with explicit visual cues (E-IMP) potentiated alpha-band strength over visual-attentional brain areas, namely frontal and parieto-occipital areas, compared to training with explicit written instructions (E; Figure 5C). As opposed to E-IMP participants, we observed that participants in the E group showed a trend for relatively decreased alpha-band power over the right temporal, left parieto-occipital, and central-frontal areas. Despite the different neurophysiological trends in each group, both groups improved their motor performance. This finding must be interpreted with care. Already at Baseline, we found that E participants showed enhanced alpha-band activity over frontoparietal areas when compared to E-IMP participants. However, at Retention, the trend was inverted, i.e., E-IMP participants showed an enhancement of alpha-band power over frontal and parieto-occipital areas compared to E participants. Therefore, our neurophysiological and behavioural data suggest that training with different task instructions (E-IMP and E) may engage different neural processes during training—visuo-attentional processes (linked to frontal and parieto-occipital regions, in the E-IMP group) and verbal-analytical processes (linked to areas, such as the right-temporal and central-frontal regions, in the E group)—, both supporting motor performance.

Finally, leaving participants to freely explore the task dynamics (IMP) led to significantly different responses after training than letting participants know some instructions about the task (E and E-IMP). The E-IMP participants enhanced the alpha-band power over left fronto-temporal regions relatively more than IMP participants. In turn, IMP participants showed a slighter decrease of alpha-band power over right frontotemporal areas when compared to E participants. This activation pattern—i.e., alpha-band power decreased over fronto-temporal areas and increased over parieto-occipital regions (Figure 5B)—lends support to the idea that both visuo-attentional and verbal-analytical processes could support learning by self-driven exploration (IMP group), typical of earlier stages of learning as seen in the relatively higher joystick variability shown by IMP group.

A broad literature corpus provides evidence about the neurophysiological implications of local alpha-band power (Haegens et al., 2011; Klimesch, 2012) and synchrony (Palva and Palva, 2007), as well as the functional link of alpha-band power and attentional/working memory processes (Palva and Palva, 2007; Klimesch, 2012). However, the interpretation regarding the functional role of increased or decreased local alpha-band power is ambiguous in the literature, and therefore, our conclusions remain speculative in that domain. This study provides, instead, new insights into the role of alpha-band topographic distributions on attention. In the light of our results, we can conclude that different event-related alpha-band topographic modulations (i.e., locally and globally increased/decreased average alpha-band power) may represent task-related changes in attentional networks linked to different instruction modalities (i.e., visual or written), which are processed in distinct neural networks, supporting participant’s perception of task-relevant stimuli and motor performance (Liao and Masters, 2001; van Duijn et al., 2020).

### Limited Behavioural and Neurophysiological Skill Transfer Observed in an Obstacle Avoidance Task

Although participants in the E-IMP group significantly increased their speed and distance surfed toward the finish line and reduced their alignment error at the OT (as also observed in HT), this ability to surf waves did not result in significant improvements in their success rate during OT, nor reduced their obstacle avoidance inability after training. The IMP group increased the distance surfed toward the finish line in OT (contrary to what we observed in HT), at the cost of reducing their success rate after training (significantly more than the E and E-IMP groups). At Retention, participants in the E group did not show any significant changes in any of the performance metrics with respect to Baseline. Overall, pre-post training changes were not significant across groups, except for the success rate metric.

Participants trained without instructions (IMP) may have prioritized reaching the finish line *(“surf as fast as possible”*) over avoiding collisions, explaining the reduced success rate of the IMP group in the OT compared with the other groups. Therefore, instructions provided using visual cues (E-IMP) and in written (E) might help in harmonizing both aims of the task: reaching the finish line as fast as possible without sacrificing the success rate in avoiding the obstacles, as opposed to training without instructions (IMP). Additionally, the OT task, by design, imposes requirements on participant’s ability to avoid obstacles (“*[…] and avoid the obstacles*”), which is lacking in the HT. Therefore, we could argue that training with instructions (E-IMP and E groups) provided a certain advantage, settling an accuracy over speed trade-off when exercising in the OT. However, all conditions were designed to equally prioritize accuracy over speed.

Similar to the HT, our neurophysiological findings suggest that task instructions modulated the pre-post engagement of average attentional networks in the obstacle task. We found nearly significant differences in the networks present in the E-IMP group pre-post training in the obstacle avoidance task on the neurophysiological level, showing a trend toward decreasing alpha-band power over bilateral frontal-lateral and parieto-occipital clusters. Such topographical distribution did not qualitatively resemble the networks observed during the HT (i.e., increased frontal and parieto-occipital alpha-band power). Instead, the decreased occipital alpha-band power could reflect active neuronal processing, for example, in visual areas (Palva and Palva, 2007), in charge of perceiving the obstacles during the OT. However, provided we only found a statistical trend, the interpretation of our findings remains limited.

Taken all together, the transfer of the skills to an obstacle avoidance task was limited. We expected that participants would exploit the learned underlying task rule (i.e., to accelerate the boat by steering toward the wave direction at incoming wave onset) to also halt the boat to avoid obstacles (assessed in the OT). However, the OT (forced decision-making reaction task, i.e., choosing between heading straight or fully misaligning the boat) was, perhaps, too different from HT (steering toward a continuum of possible direction angles, i.e., a planning task) and the training tasks (steering toward floating buoys with enough time to plan, i.e., a planning task), preventing transfer of the acquired skills. McDougle and Taylor (2019) have shown that the distance between trained and untrained target positions can predict the angular error w.r.t. untrained positions. Therefore, the differences between the training and HT tasks with respect to the OT, and not the different instructions used during the training phase, might have determined the low transfer of skills between the different tasks.

### Study Limitations and Future Research Opportunities

In this study, we observed differences in behavioural and neural changes that are related to providing vs. not providing task instructions while practicing a motor task on a relatively small participants’ sample size (i.e., 12 participants per group). The inter-individual variability in our kinematic data might have prevented us from detecting significant between-group differences in more metrics. Thus, studies with higher sample sizes are needed to make more sensitive analyses. In addition, more training sessions and long-term retention tests are necessary to study the effect of different instructions on attention and motor performance at later stages of motor learning, i.e., at the autonomous stage (Fitts, 1964).

Although we chose to show visual cues to all participants, regardless of the group they were allocated to, participants in the E-IMP group were presented with different visual stimuli: the buoy colour was either green or red (instead of a random colour) and had a meaning (namely, to avoid or catch a buoy) that for this group would also result on missing or catching the wave, as the buoys were placed close in front of the boat and only in the wave direction. On the one hand, the buoys provided information about the correct alignment to catch a wave. On the other hand, training to avoid a wave would, perhaps, provide richer information about the boat-wave interaction (e.g., compared to always catching a wave). Therefore, participants in the E-IMP group may have experienced a type of training that resulted in increased saliency of the wave compared to participants in the E and IMP groups. As such, the similarity between the E-IMP training (i.e., catching or avoiding buoys) and the OT task (i.e., avoiding appearing obstacles) could also explain why E-IMP participants showed mild behavioural and neurophysiological pre-post training changes in the obstacle avoidance task (OT). However, the similarity between the OT and the E-IMP training task did not result in a general advantage on the participants’ performance in OT when compared to the E and IMP groups.

The alpha-band frequency ranges (7–15 Hz) chosen for our analyses overlap with two potentially confounding signals present in human EEG: spindles and mu-rhythms. The spindles—an increased spontaneous (non-time-locked) daytime global alpha-band oscillation in the 7–15 Hz spectral band—are thought to reflect mental fatigue (Lopes da Silva, 1991; Klimesch, 2012). Because we focused on time-locked (i.e., event-related) modulations of alpha-band oscillations, the continuous spontaneous alpha-band activity should be canceled out in our analyses, making it implausible that our observations reflect mental fatigue. Further, the mu-rhythms—commonly observed during motor preparation and execution strictly over electrodes neighbouring the hand knob area at the central sulcus (∼10 Hz; Pfurtscheller, 1992; Pfurtscheller et al., 1996)—are also within the frequency range of interest used in this study. However, our analyses show an event-related topographical modulation of the alpha-band oscillations occurring around incoming wave onset, unlike mu-rhythms registered several seconds after cue onset (Pfurtscheller et al., 1996). Therefore, our findings likely reflect attention-mediated alpha-band modulations rather than motor- or fatigue-related neural activity.

Our novel stimulus-response paradigm aims to be closer to real-life than simpler stimulus-response tasks. However, this novelty comes with few limitations: potential subject-specific strategies might explain the variability observed in our results, the need to come up with a task-specific behavioural analysis, difficulties in obtaining a strong statistical effect size, or the presence of other potential confounding factors, such as movement artifacts in the EEG signal. We tried to overcome these limitations associated with the complexity of our paradigm via careful study design.

Although we tried to control the speed-accuracy trade-off among groups during training with our instruction design, it is still possible that inter-individual differences influenced how participants prioritized speed vs. accuracy or other strategy-related factors, e.g., surfing style or risk-taking character. We could not appreciate a generalization of the skills acquired during training to another virtual task: the obstacle avoidance task. Further experiments that control the variety of potential strategies that participants can follow to fulfil the tasks (both in HT and OT) could complement and confirm our contribution despite reducing the ecological validity.

Although we selected a complex visuomotor task that resembles real-life surfing, the task is still a lab-based virtual paradigm, which misses important task-related sensory information from real-life settings (e.g., vestibular and haptic sensory information) (Özen et al., 2020). Moreover, participants’ hand movements in our paradigm were rather small to avoid EEG artifacts. Therefore, the direct transfer of our findings to a real-life setting is limited. Nevertheless, our lab-based virtual simulation of a real-life task has important applications in motor training. In our study, we found that providing instructions that enforce the task rules may be the best approach to enhance participants’ motor performance. However, how the underlying task rule is enforced seems to be secondary. For example, task rules could be enforced by instructions provided prior to training (e.g., coaches could help trainees focusing their attention on task-relevant stimuli, typically using explicit instructions), but also presented during training using visual cues (e.g., using commercial virtual and augmented reality displays).

These findings are of great interest for the design of better training routines in real-life scenarios where task instructions and/or visual cues may be employed, e.g., sports training, surgical training, and simulator-based education. Additionally, the novel task design employed in our study is proof of the feasibility of the application of complex real-life visuomotor tasks to measure event-related neural activity. Thus, we encourage researchers to explore well-known neural correlates in real-life paradigms in order to assess the transferability of basic neuroscience principles into real-life scenarios, bridging the gap between the lab and real-life settings. Finally, the alpha-band spatio-temporal dynamics pre-post training could be exploited, for example, to discriminate between poor and good performers. Thus, explicit knowledge and visual cues enforcing the task rule could be provided during training, depending on the trainees’ visuo-attentional or working memory capacities, to support motor learning to optimally support motor learning.

## Conclusion

In conclusion, to the best of our knowledge, this study is the first to investigate the effect of task instructions about the underlying task rule on motor performance and neural correlates of attention. We found that providing task instructions seems to boost learning of a virtual surfing task, compared to letting participants surf freely. Nevertheless, providing task instructions in written or using visual cues leads to similar improvements in performance by relying on different attentional networks. On a neurophysiological level, we found differences between explicit visual cueing linked to (top-down) visuo-attentional processes and explicit written commands related to verbal-analytical processes, compared to the activation pattern observed after training the task without instructions. Although our findings do not have a direct implication for surfing/sailing training, the results of our work contribute to gaining a better understanding of the neural and behavioural effects of enforcing underlying task rules using visual cues or written commands in applications where instructions are commonly used to improve motor (re)learning, such as sports training and neurorehabilitation.

## Data Availability Statement

The datasets presented in this study can be found in online repositories. The names of the repository/repositories and accession number(s) can be found in the article/Supplementary Material.

## Ethics Statement

The studies involving human participants were reviewed and approved by Kantonale Ethikkommission Bern (KEK), (ref.: 2018-01179) and the Swiss Agency for Therapeutic Products (Swissmedic ref.: 10000432). The study is registered in ClinicalTrials.gov (NCT04759976) and EUDAMED (CIV-19-01-026764) under the title “Optimize motor Learning to Improve Neurorehabilitation” (“OnLINE”). The patients/participants provided their written informed consent to participate in this study.

## Author Contributions

JP-A, KB, and LM-C designed the study, wrote the manuscript, and analysed the behavioural dataset. JP-A set up the experiment, designed and programmed the motor task. JP-A and KB collected the data. JP-A, KB, and TK analysed the electrophysiological dataset. All authors edited and revised the manuscript and approved the submitted version.

## Conflict of Interest

The authors declare that the research was conducted in the absence of any commercial or financial relationships that could be construed as a potential conflict of interest.

## Publisher’s Note

All claims expressed in this article are solely those of the authors and do not necessarily represent those of their affiliated organizations, or those of the publisher, the editors and the reviewers. Any product that may be evaluated in this article, or claim that may be made by its manufacturer, is not guaranteed or endorsed by the publisher.

## Footnotes

1
https://github.com/wave-harmonic/crest
