# Supplementary material for: Providing Task Instructions During Motor Training Enhances Performance and Modulates Attentional Brain Networks
Source: Front Neurosci. 2021 Dec 9;15:755721. doi: 10.3389/fnins.2021.755721 (PMC8695982; doi:10.3389/fnins.2021.755721)
Supplement: Supplementary file 2 [file Image_1.pdf]

Supplementary Material

1 Supplementary Figures

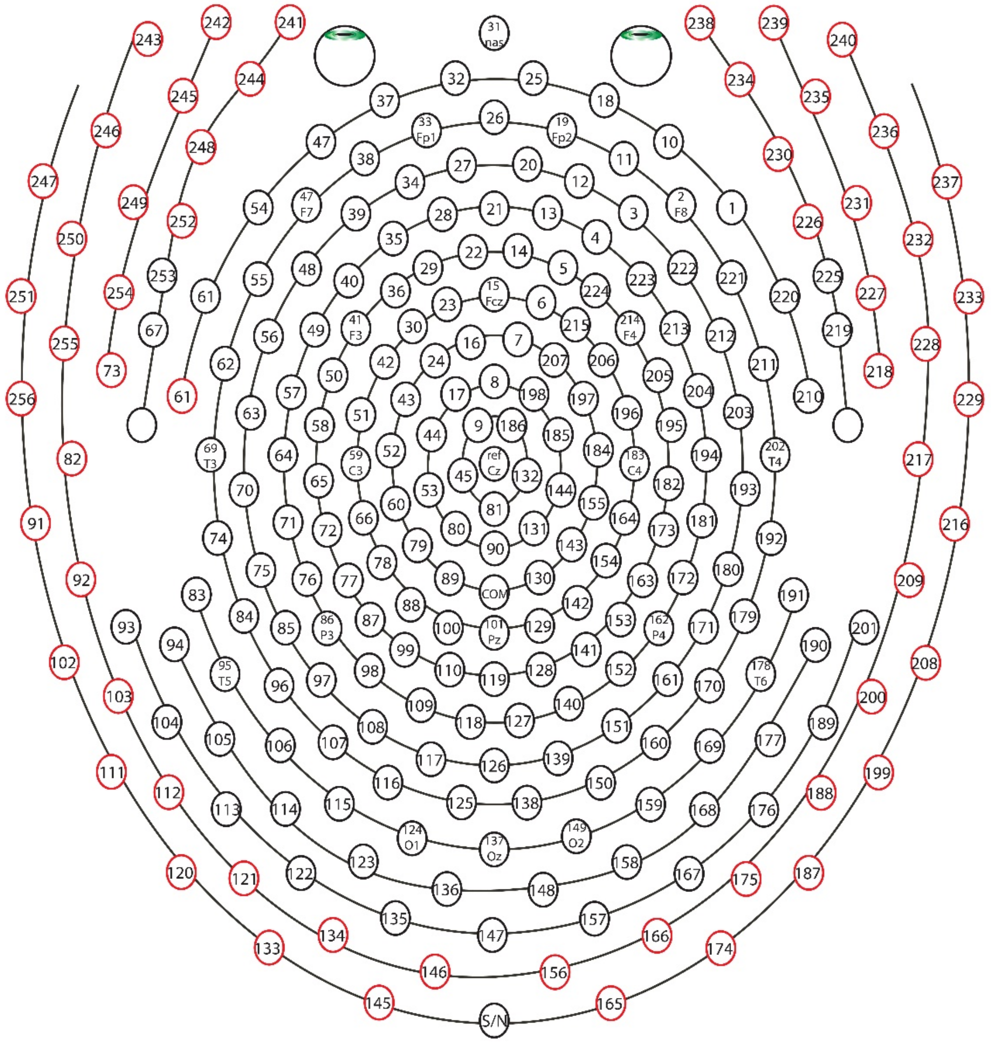

Supplementary Figure 1. Position of electrodes in the 256-channel Hydrogel cap (*Electric Geodesics, USA*). In red, electrodes discarded due to the low signal-to-noise ratio
